# Loss of p53-mediated cell-cycle arrest, senescence and apoptosis promotes genomic instability and premature aging

**DOI:** 10.18632/oncotarget.7864

**Published:** 2016-03-02

**Authors:** Tongyuan Li, Xiangyu Liu, Le Jiang, James Manfredi, Shan Zha, Wei Gu

**Affiliations:** ^1^ Institute for Cancer Genetics, and Department of Pathology and Cell Biology, College of Physicians and Surgeons, Columbia University, New York, NY, USA; ^2^ Herbert Irving Comprehensive Cancer Center, College of Physicians and Surgeons, Columbia University, New York, NY, USA; ^3^ Department of Oncological Sciences, Icahn School of Medicine at Mount Sinai, New York, NY, USA; ^4^ Department of Pediatrics, College of Physicians and Surgeons, Columbia University, New York, NY, USA

**Keywords:** p53, acetylation, ferroptosis, tumor suppression, genomic instability

## Abstract

Although p53-mediated cell cycle arrest, senescence and apoptosis are well accepted as major tumor suppression mechanisms, the loss of these functions does not directly lead to tumorigenesis, suggesting that the precise roles of these canonical activities of p53 need to be redefined. Here, we report that the cells derived from the mutant mice expressing p53^3KR^, an acetylation-defective mutant that fails to induce cell-cycle arrest, senescence and apoptosis, exhibit high levels of aneuploidy upon DNA damage. Moreover, the embryonic lethality caused by the deficiency of XRCC4, a key DNA double strand break repair factor, can be fully rescued in the p53^3KR/3KR^ background. Notably, despite high levels of genomic instability, p53^3KR/3KR^XRCC4^−/−^ mice, unlike p53^−/−^ XRCC4^−/−^ mice, are not succumbed to pro-B-cell lymphomas. Nevertheless, p53^3KR/3KR^ XRCC4^−/−^ mice display aging-like phenotypes including testicular atrophy, kyphosis, and premature death. Further analyses demonstrate that SLC7A11 is downregulated and that p53-mediated ferroptosis is significantly induced in spleens and testis of p53^3KR/3KR^XRCC4^−/−^ mice. These results demonstrate that the direct role of p53-mediated cell cycle arrest, senescence and apoptosis is to control genomic stability *in vivo*. Our study not only validates the importance of ferroptosis in p53-mediated tumor suppression *in vivo* but also reveals that the combination of genomic instability and activation of ferroptosis may promote aging-associated phenotypes.

## INTRODUCTION

Given the importance of p53 in tumor development, genomic integrity and normal aging process, p53 activities are tightly regulated at multiple levels by a delicate network through various positive/negative regulators, cofactors, and a large number of posttranslational modifications, including phosphorylation, ubiquitination and acetylation [1-5]. Accumulated evidence shows that acetylation is essential for p53 activation and plays an important role in regulating promoter-specific activation of p53 target genes in response to various stress signals [6]. In addition to the six lysine (K) residues clustered at the C-terminal domain, we and others identified additional acetylation sites of p53, K120 and K164, situated within the DNA-binding domain. K120 and K164 are highly conserved throughout evolution and crucial for p53-medited apoptosis and cell cycle arrest respectively [7-9]. To understand the *in vivo* roles of p53 acetylation, we previously generated the p53^3KR/3KR^ knock-in mouse model in which three corresponding acetylation sites (K117, K161 and K162 in mouse p53) were mutated to the non-acetylable arginine [10]. While loss of acetylation at these sites completely abrogated p53-mediated cell cycle arrest, apoptotic cell death and cellular senescence, p53^3KR/3KR^ mice do not succumb to spontaneous tumors as documented for previous reported p53^−/−^ mice [11, 12], indicating that loss of p53-mediated acute DNA damage response is not sufficient for tumorigenesis [10]. Studies of other mouse models, including p53^25,26^ and p21^−/−^Puma^−/−^Noxa^−/−^ also suggested that p53-mediated tumor suppression activity cannot be solely attributed to these well known targets of p53 in stress responses [13, 14]. Taken together, these studies imply that other mechanisms are critical for p53 to exert its tumor suppressor function *in vivo*.

Numerous studies show that p53 plays a critical role in the maintenance of genomic integrity through its role in DNA damage responses [3, 15]. Loss of p53 function promotes chromosomal instability and wild-type p53 functions as a hub of DNA damage activated checkpoints and as a barrier against genomic instability [16, 17]. In this context, p53 deficiency rescues embryonic lethality caused by the inactivation of many DNA double stand break(DSB) repair genes, such as XRCC4, by attenuating cell cycle checkpoint control, apoptosis and senescence to allow cell survival with genomic instabilities [18-21]. The inherent drawbacks of these rescues are often early onset lethal tumors, which preclude long term studies. The p53^3KR/3KR^ mutant fail to induce p53-mediated cell cycle arrest, apoptotic cell death and senescence in response to DNA damage, yet still retains tumor suppression capacities, providing a unique tool to dissect the mechanisms of p53-mediated activities *in vivo* [10]. As such, *p53^3KR/3KR^Xrcc4^−/−^ m*ice were generated by crossing p53^3KR^ and XRCC4 DNA repair-impaired mutants, and here we show that p53^3KR/3KR^ mutant completely rescue the embryonic lethality caused by XRCC4 deficiency, and unlike p53^−/−^ Xrcc4^−/−^ mice, p53^3KR/3KR^ Xrcc4^−/−^ mice show a strong resistance to tumor formation, but surprisingly, display the accelerated aging phenotypes. We further showed that p53-mediated ferroptosis is significantly induced in both spleens and testis of p53^3KR/3KR^XRCC4^−/−^ mice. These results redefine the role of p53-mediated cell-cycle arrest, senescence and apoptosis and have significance implications for the roles of p53-mediated ferroptosis *in vivo*.

## RESULTS

### p53^3KR/3KR^ cells display high levels of aneuploidy in response to DNA damage treatment

**Figure 1:**
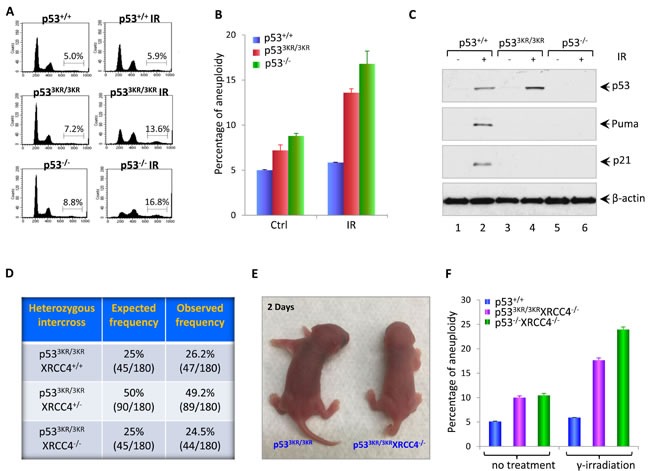
Loss of p53-mediated acute DNA damage response causes genomic instability **A.** Flow cytometric analysis of cell cycle distribution in p53^+/+^, p53^3KR/3KR^, and p53^−/−^ MEFs. MEFs were either left untreated or exposed to 10 Gy of γ-irradiation; 24 hours later, MEFs were collected and fixed with 70% ethanol for 1hour at 4°C, then subjected to FACS after propidium iodide (PI) staining. **B.** Quantification of the percentage of MEFs with aneuploidy. Error bars represent averages ± SD from at least three independent MEF lines for each genotype. **C.** Western blot analysis of p53^+/+^, p53^3KR/3KR^ and p53^−/−^ MEFs. Cells were either untreated or exposed to 10 Gy of γ-irradiation, then lyzed and analyzed for the expression of p53, p21, and Puma. β-actin was used as a loading control. **D.** Table showing the expected and observed frequency from the intercross of p53^3KR/3KR^Xrcc4^+/−^ mice. **E.** Representative pictures of p53^3KR/3KR^XRCC4^−/−^ mice and p53^3KR/3KR^ littermates at 2 days of age. **F.** The percentage of aneuploidy by FACS analysis of cell cycle distribution in p53^+/+^, p53^3KR/3KR^XRCC4^−/−^, and p53^−/−^XRCC4^−/−^ MEFs. Cells were either left untreated or exposed to 10 Gy of γ-irradiation. 24 hours post-radiation, MEFs were collected, fixed with 70% ethanol for 1hour at 4°C, and then subjected to FACS after propidium iodide (PI) staining. Data are shown as averages ± SD from three independent MEF lines for indicated genotypes.

Aneuploidy caused by cell division errors is one of the most common types of genomic instabilities found in human cancers [22]. The increased level of aneuploidy was frequently observed in cells from *p53^−/−^* mice, which exhibited high levels of genomic instability and early onset thymic lymphomas with aneuploidy [23-25]. So we first examined the aneuploidy level in *p53^3KR/3KR^* MEFs. DNA content analysis by FACS shows that primary *p53^3KR/3KR^* MEFs at passage 1 (P1) have a slightly higher basal level of aneuploidy compared with WT MEFs (P1) (Figure 1A and Figure 1B). In response to ionizing radiation (IR), p53-mediated transactivation of *Puma* and *p21* are completely abrogated in p53^3KR/3KR^ MEFs as shown in Figure 1C, however, unlike WT MEFs, *p53^3KR/3KR^* MEFs exhibit an increased level of aneuploidy 24 hours post-radiation, which is comparable to *p53^−/−^* MEFs (Figure 1A and 1B), suggesting that the *p53^3KR/3KR^* MEFs is prone to radiation-induced aneuploidy.

### The embryonic lethality caused by the deficiency of XRCC4 can be fully rescued in the p53^3KR/3KR^ background

In normal cells, the genome integrity is constantly challenged by inevitable DNA lesions often arising as byproducts of normal cellular processes such as reaction oxygen species or DNA replication stress, leading to DSBs in chromosome; unrepaired DNA DSBs can activate DNA damage responses and induce p53 activation [26, 27]. Homologous recombination (HR) and non-homologous end-joining (NHEJ) are two major DNA DSB repair pathways in mammalian cells [28]. XRCC4 is essential for the protein stability of Ligase 4 - the DNA ligation component of the NHEJ pathway, which is also required for V(D)J recombination in developing lymphocytes. XRCC4-deficient embryos are growth-retarded and die at embryonic day 15.5 with massive p53-mediated neuronal apoptosis [29, 30]. While p53 deficiency full resuced the embryonic lethality of Xrcc4^−/−^ mice, p53^−/−^Xrcc4^−/−^ mice routinely succumb to pro-B-cell lymphomas and medulloblastomas [19, 21]. To investigate the genomic instability caused by loss of p53-mediated cell cycle arrest, apoptosis, and senescence *in vivo*, we crossed *p53^3KR^* mice with XRCC4 mutant mice and eventually obtained *p53^3KR/3KR^Xrcc4^−/−^* mice from breedings between *p53^3KR/3KR^Xrcc4^+/−^* mice. *p53^3KR/3KR^Xrcc4^−/−^* mice were born at the expected Mendelian ratio (44 out of 180), indicating *p53^3KR/3KR^* fully rescues the embryonic lethality caused by XRCC4 deficiency (Figure 1D). *p53^3KR/3KR^Xrcc4^−/−^* mice are morphologically normal but slightly smaller than *p53^3KR/3KR^Xrcc4^+/+^* mice at birth (Figure 1E). To examine the genomic instability, we first measured aneuploidy in *p53^3KR/3KR^Xrcc4^−/−^* MEFs together with WT and *p53^−/−^Xrcc4^−/−^* control MEFs. MEFs were either left untreated or exposed to 10 Gy γ-irradiation and analyzed 24 hours post-radiation. FACS analyses of cell cycle distribution using DNA content measurement revealed that the percentage of cells with aneuploidy in *p53^3KR/3KR^Xrcc4^−/−^* MEFs (10%) is similar to that in *p53^−/−^Xrcc4^−/−^* MEFs (10.5%), but doubled in comparison with WT MEFs (5.1%). In contrast to the WT MEFs that had similar aneuploidy percentage before and after 10 Gy IR treatments, indicative of intact DNA damage responses, *p53^3KR/3KR^Xrcc4^−/−^* MEFs, similar to *p53^−/−^Xrcc4^−/−^* MEFs, exhibited further augmented aneuploidy levels 24 hours after γ-irradiation (Figure 1F). Taken together, these results demonstrate that the embryonic lethality of *Xrcc4^−/−^* mutant mice can be completely rescued in the *p53^3KR/3KR^* background. A markedly increase in aneuploidy of *p53^3KR/3KR^Xrcc4^−/−^* cells suggests that the loss of p53-mediatded cell cycle arrest, apoptosis and senescence leads to genomic instability.

### Spontaneous genomic instability in *p53^3KR/3KR^Xrcc4^−/−^* mice

**Figure 2:**
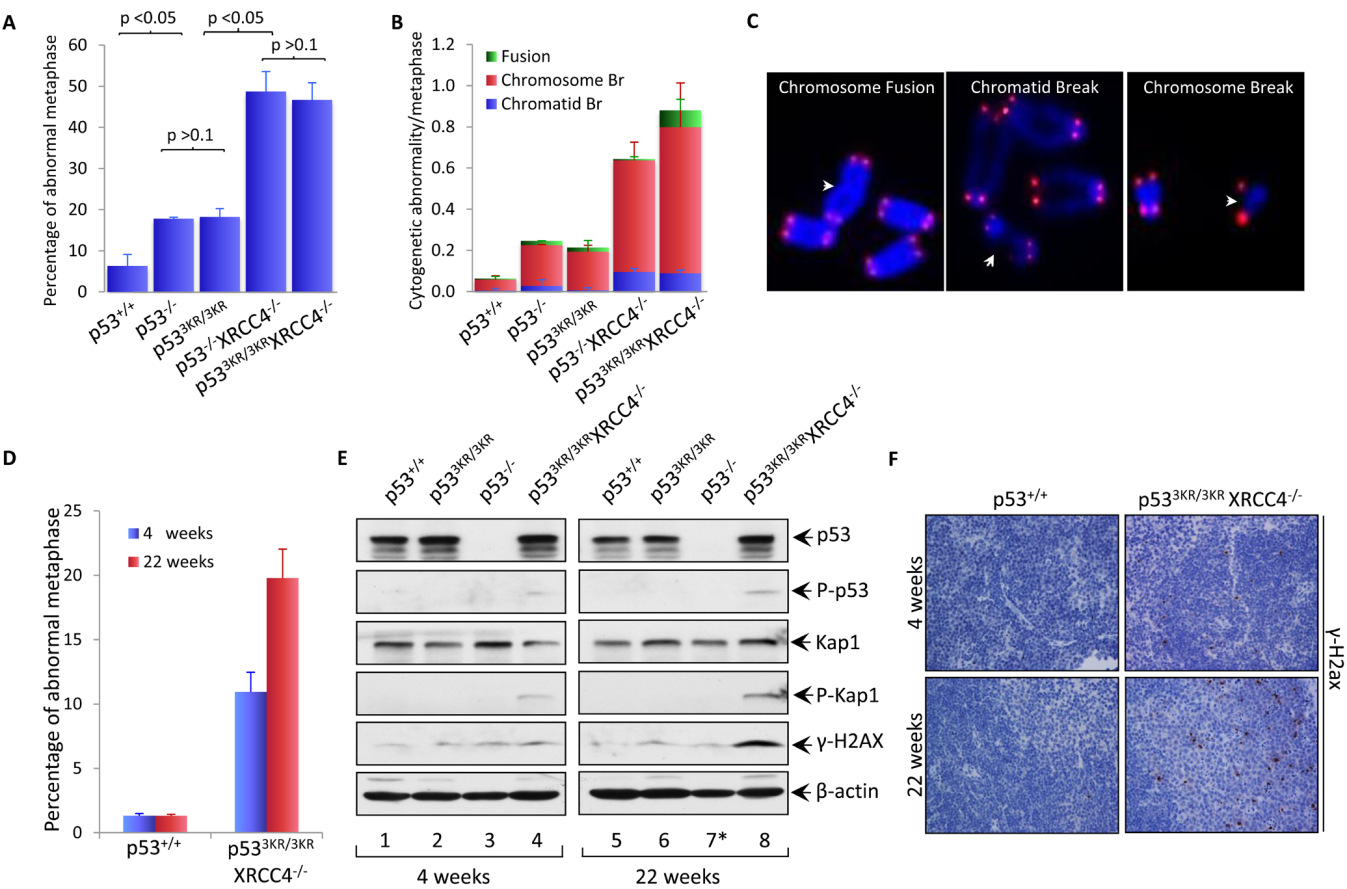
Spontaneous genomic instability in p^533KR/3KR^XRCC4^−/−^ mice was augmented with age **A.** Percentage of abnormal metaphase spreads obtained from early passage p53^+/+^, p53^3KR/3KR^, p53^−/−^, p53^3KR/3KR^XRCC4^−/−^, and p53^−/−^XRCC4^−/−^ MEFs. Metaphases were prepared from MEFs for the indicated phenotypes after 3 hours treatment with 0.1μg/ml colcemid and analyzed using telomere-FISH assay. At least 100 metaphases were counted. Results are shown as averages ± SD from three different MEF lines of each genotype. **B.** Frequency of cytogenetic abnormalities in three categories: chromosomal breaks, chromatid breaks and chromosomal fusions, in MEFs with the indicated genotypes. Values shown are the averages ± SD from three independent MEF lines. **C.** Representative images of abnormalities observed in p53^3KR/3KR^XRCC4^−/−^ MEF metaphases. Chromosomes were stained with telomere specific probes (red) and counterstained with DAPI (blue).**D.** Percentage of metaphases with abnormalities in bone marrows from 4-week-old and 22-week-old p53^+/+^ and p53^3KR/3KR^XRCC4^−/−^ mice. Metaphase spreads were obtained from mice bone marrows with the indicated genotypes after with incubation with 0.1μg/ml colcemid for 6 hours and a minimum of 100 metaphases was analyzed for each sample using telomere-FISH. Results are reported as averages ± SD from three mice for each genotype. **E.** Immunoblot assays of p53, phopho-p53, Kap1, phopho-Kap1, γ-H2ax and β-actin proteins in the lysates prepared from the spleens of p53^+/+^, p53^3KR/3KR^, and p53^−/−^ p53^3KR/3KR^XRCC4^−/−^ mice at the indicated ages. *; The 3-month-old p53^−/−^ mice (lane 7) was used for control. β-actin was used as a loading control. **F.** Representative immunohistochemical staining of spleens from 4 week- and 22 week-old p53^+/+^ and p53^3KR/3KR^XRCC4^−/−^ mice for γ-H2ax.

To further characterize the spontaneous genomic instability observed in *p53^3KR/3KR^Xrcc4^−/−^* MEFs, we obtained metaphase spreads from early passage (P1) *p53^3KR/3KR^Xrcc4^−/−^* MEFs as well as WT, *p53^3KR/3KR^*, *p53^−/−^* and *p53^−/−^Xrcc4^−/−^* control MEFs and quantified chromosomal breaks with telomere-fluorescence *in situ* hybridization (T-FISH) analysis [31, 32]. As shown in Figure 2A and Figure S1, overall percentage of abnormal metaphases in *p53^3KR/3KR^* MEFs (18.2%) is similar to that in *p53^−/−^* MEFs (17.8%), in contrast to only 6.3% of *p53^+/+^* MEFs. XRCC4 deficiency dramatically increased the frequency of abnormal metaphases in both *p53^−/−^* and *p53^3KR/3KR^* background to 48.7% and 46.7%, respectively. T-FISH can identify three types of cytogenetic aberrations, namely chromatid breaks - breaks at one of two sister chromatids, chromosome breaks - breaks at both sister chromatids and chromosomal fusion - joining of two chromosome ends in the absence of telomere signals (Figure 2C). Chromosomal translocations without altering telomere signal cannot be unequivocally identified in T-FISH analyses, leading to underestimation of chromosomal fusion by T-FISH assay. Chromosome breaks, chromatid breaks and chromosome fusion were increased significantly in *p53^3KR/3KR^Xrcc4^−/−^* MEFs as seen in *p53^−/−^Xrcc4^−/−^* MEFs (Figure 2B and Figure S2), validating the genomic instability in those cells. Moreover, Western blot analyses revealed that several DNA damage markers such as phospho-ATM, phospho-p53, phospho-Kap1 and phospho-H2AX (γ-H2AX) were readily detected in *p53^3KR/3KR^Xrcc4^−/−^* MEFs (lane 5, Figure S3).

To assess the levels of spontaneous genomic instability observed *in vivo*, we isolated bone marrow from 4 weeks and 22 weeks old *p53^3KR/3KR^Xrcc4^−/−^* mice using age-matched *p53^+/+^* mice as controls. Metaphase spreads were prepared from total bone marrows after incubation with 0.1 μg/ml colcemid for 6 hours and subjected to T-FISH analyses. As shown in Figure 2D, the percentage of abnormal metaphases in *p53^3KR/3KR^Xrcc4^−/−^* bone marrow cells increased significantly from 10.9% at 4 weeks to 19.8% at 22 weeks, whereas abnormal metaphase counts remain steady in *p53^+/+^* mice at the same age period (also see Figure S4). We also examined the levels of proteins involved in the maintenance of genome stability in the spleens of *p53^3KR/3KR^Xrcc4^−/−^* mice at 4 weeks and 22 weeks of age by Western blot analyses. As shown in Figure 2E, phospho-p53, phospho-Kap1 and γ-H2AX were readily detected in splenocytes from *p53^3KR/3KR^Xrcc4^−/−^* mice at 4 weeks of age whereas none of them was detected in the splenocytes of *p53^+/+^*, *p53^3KR/3KR^* and *p53^−/−^* mice at the same age. Moreover, the levels of these DNA damage markers were further increased in *p53^3KR/3KR^Xrcc4^−/−^* mice at 22 weeks of age compared with age-matched control mice. Immunohistochemical analyses also confirmed a marked increase of the percentage of γ-H2AX positive cells in the spleens of *p53^3KR/3KR^Xrcc4^−/−^* mice at 22 weeks of age (Figure 2F). Taken together, these data demonstrate spontaneous genomic instability in *p53^3KR/3KR^Xrcc4^−/−^* mice.

### The *p53^3KR/3KR^Xrcc4^−/−^* mice are not predisposed to cancer but nevertheless have a short life-span

**Figure 3:**
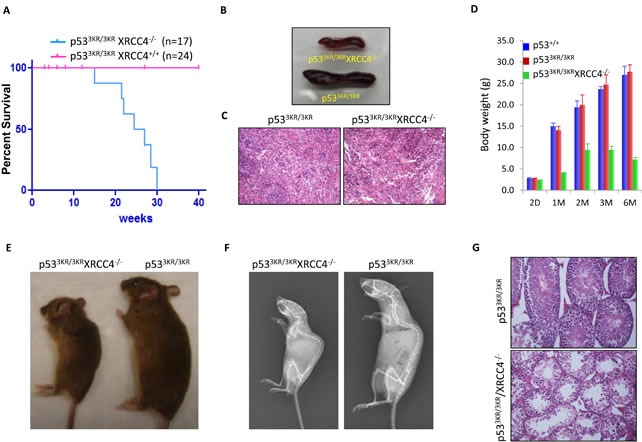
p^533KR/3KR^XRCC4^−/−^ mice bearing high levels of genomic instability are resistant to cancer but display premature aging phenotypes **A.** Kaplan-Meier overall survival curves of p53^3KR/3KR^ and p53^3KR/3KR^XRCC4^−/−^ mice. **B.** Images of spleens of p53^3KR/3KR^XRCC4^−/−^ mouse and p53^3KR/3KR^ littermate at the age of 22 weeks.**C.** Hematoxylin and eosin (H&E) staining of spleens from 22-week-old p53^3KR/3KR^ and p53^3KR/3KR^XRCC4^−/−^ mice.**D.** Average body weight of p53^+/+^, p53^3KR/3KR^, and p53^3KR/3KR^XRCC4^−/−^ mice at different ages. Error bars means averages ± SD. **E.** p53^3KR/3KR^XRCC4^−/−^ mouse and p53^3KR/3KR^ littermate at 22 weeks of age.**F.** Representative whole-body radiographs of 22-week-old p53^3KR/3KR^XRCC4^−/−^ mice with p53^3KR/3KR^ littermates. **G.** H&E staining of testis from 22-week-old p53^3KR/3KR^ and p53^3KR/3KR^XRCC4^−/−^ mice.

The above data indicate that *p53^3KR/3KR^Xrcc4^−/−^* mice show high levels of spontaneous genomic instability, but unlike *p53^−/−^Xrcc4^−/−^* mice that uniformlly succumb to pro-B-cell lymphomas by ∼10 weeks [19], *p53^3KR/3KR^Xrcc4^−/−^* mice lived up to 30 weeks (Figure 3A). Since *p53^−/−^Xrcc4^−/−^* mice mainly succumb to pro-B-cell lymphomas, we performed the further analyses of the spleens. The spleens of *p53^3KR/3KR^Xrcc4^−/−^* mice appear normal but smaller compared to the age-matched control mice (Figure 3B), likely due to the absence of mature B and T cells caused by Xrcc4 deficiency. Histological examinations of spleens of *p53^3KR/3KR^Xrcc4^−/−^* mice showed no evidence of lymphomas, indicated by infiltration of clonal enlarged B/T cells (Figure 3C). Further analysis also failed to identify any tumors in other organs of *p53^3KR/3KR^Xrcc4^−/−^* mice. *p53^3KR/3KR^Xrcc4^−/−^* newborn mice exhibit slight differences in overall body weight compared to their littermates (Figure 3D). By 4 weeks of age, their body weight was more than 70% lower than control littermates. *p53^3KR/3KR^Xrcc4^−/−^* mice reach their maximum body weight at 8 weeks of age, which is only about 50% normal control mice, and then started weight loss (Figure 3D). Interestingly, the whole body X-ray analysis showed that *p53^3KR/3KR^Xrcc4^−/−^* mice displayed kyphosis (also known as hunchback), an abnormal curvature of the spine, at the age of 22 weeks (Figures 3E and 3F), one of the main premature aging associated phenotypes that were previously reported in p53 mutant mice [33, 34].

We next sought to examine the possibility that the shortened lifespans in *p53^3KR/3KR^Xrcc4^−/−^* mice could be accompanied by more defined premature aging phenotypes in addition to aging-associated kyphosis. Aging related alterations in the male reproductive system occur primarily in the testis. The mammalian testis is a complex organ composed of germ cells and supporting cells such as Leydig cells and Sertoli cells that together produce functional spermatozoa and hormones respectively through a series of orchestrated developmental transitions in the seminiferous tubules. It has been reported that the production of mature sperms and their quality gradually decline with age in humans and studies using mouse models have also shown convincing evidence that the proliferation and maturation of germ cells can become compromised with aging, leading to testicular atrophy [35]. *p53^3KR/3KR^Xrcc4^−/−^* mice can live up to about 30 weeks but they are all sterile. Histological examinations of testes of 22-week-old *p53^3KR/3KR^Xrcc4^−/−^* mice revealed a marked reduction of testicular mass, extensive seminiferous epithelium degeneration and severe tubule atrophy in comparison with their control littermates (Figure 3G). In the meantime, spermatogonia were not found in the tubular lumina and significant fractions of the spermatogonia is missing from the basal layer, consistent with the fact that spermatogenesis was completely compromised in *p53^3KR/3KR^Xrcc4^−/−^* mice (Figure 3G). Taken together, these data indicate that that *p53^3KR/3KR^Xrcc4^−/−^* mice, unlike *p53^−/−^Xrcc4^−/−^* mice, do not succumb to pro-B-cell lymphomas but have a short life span and exhibit aging-associated kyphosis and testicular atrophy at the age of 22 weeks.

### Mechanistic insights into the phenotypes observed in *p53^3KR/3KR^Xrcc4^−/−^* mice

**Figure 4:**
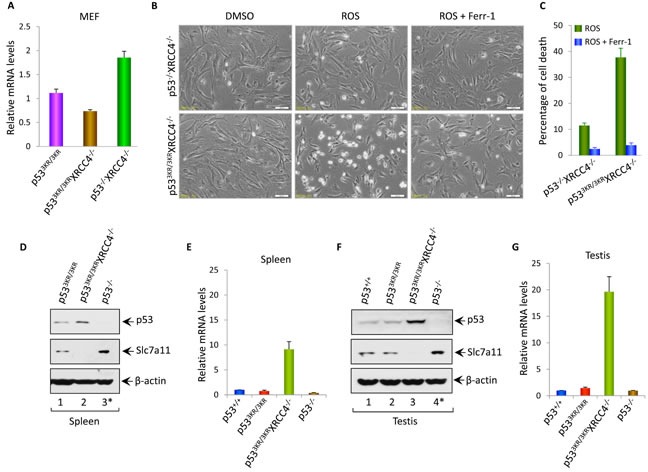
The roles of p53-mediated effects on Slc7a11 and ferroptosis in tumor suppression and aging associated testicular atrophy in p^533KR/3KR^XRCC4^−/−^ mice **A.** qRT-PCR analysis of Slc7a11 mRNA levels in p53^3KR/3KR^, p53^3KR/3KR^XRCC4^−/−^ and p53^−/−^XRCC4^−/−^ MEFs. Data were shown as average ± SEM from three independent MEF lines.**B.** p53^3KR/3KR^XRCC4^−/−^ and p53^−/−^XRCC4^−/−^ MEFs were either left untreated or exposed to ROS (Tert-butyl-hydroperoxide; 400 μM) and ferroptosis inhibitor, ferrostatin-1 (ferr-1, 2 μM) for 8 hours and then representative phase-contrast images were taken.**C.** Quantification of cell death of MEFs after the treatment with ROS and ferr-1. Data were shown as average ±SD from three independent MEF lines.**D.** and **F.** Western blot analysis of p53, Slc7a11 and β-actin proteins in the lysates prepared from the spleens (D) or testis (F) of p53^+/+^, p53^3KR/3KR^, p53^3KR/3KR^XRCC4^−/−^, and p53^−/−^ mice at the age of 22 weeks. *; The 3-month-old tumor-free p53^−/−^ mice were used for control.**E.** and **G.** qRT-PCR analysis of Ptgs2 mRNA levels in spleens (E) or in testis (G) of 22-week-old p53^+/+^, p53^3KR/3KR^, p53^3KR/3KR^XRCC4^−/−^ and 3-month-old p53^−/−^ mice. Error bars represent averages ± SEM of three mice for each genotype.

Although p53-mediated cell cycle arrest, senescence and apoptosis are well accepted as major tumor suppression mechanisms, accumulating evidence indicates that the loss of these canonical functions does not directly lead to tumorigenesis. Our recent studies show that p53-mediated regulation of ferroptosis is a new mechanism of tumor suppression that acts independently of the canonical p53 functions in cell-cycle arrest, senescence and apoptosis through suppressing SLC7A11 expression [36]. Thus, although p53^3KR^ is defective in cell-cycle arrest, senescence and apoptosis, it is very likely that *p53^3KR/3KR^Xrcc4^−/−^* mice do not succumb to pro-B-cell lymphomas by activating p53^3KR^ mediated ferroptosis. To this end, we first examined whether SLC7A11 is downregulated in *p53^3KR/3KR^Xrcc4^−/−^* MEF cells. As shown in Figure 4A, q-PCR analysis revealed a significant downregulation of SLC7A11 in *p53^3KR/3KR^Xrcc4^−/−^* MEFs compared with the control *p53^−/−^Xrcc4^−/−^* MEFs (also see Figure S5). Consistent with these results, *p53^3KR/3KR^Xrcc4^−/−^* MEF cells are very sensitive to ferroptosis upon ROS treatment whereas no obvious cell death were detected in the *p53^−/−^Xrcc4^−/−^* MEFs under same conditions (Figure 4B). As expected, ROS-induced cell death was completely rescued in the presence of ferrostatin-1, a specific inhibitor for ferroptosis [36] (Figure 4B and Figure 4C).

To provide the *in vivo* evidence to support the role of p53-mediated ferroptosis to prevent the development of pro-B-cell lymphomas, we performed the analysis of p53-mediated activities in spleens. Western blot analysis showed that p53 is stabilized in the spleens of *p53^3KR/3KR^Xrcc4^−/−^* mice, the levels of SLC7A11 are significantly downregulated (lane 2, Figure 4D). Of note, since most *p53^−/−^Xrcc4^−/−^* mice become moribund by 10 weeks due to pro-B-cell lymphomas [19, 37], we used the tumor-free *p53^−/−^* mice at the age of 3 months as a control. Since recent studies identified up-regulation of *PTGS2* as a potential molecular marker of ferroptosis [38], we examined the levels of PTGS2 expression in the spleen of *p53^3KR/3KR^Xrcc4^−/−^* mice. As shown in Figure 4E, *Ptgs2* was indeed significantly upregulated in *p53^3KR/3KR^Xrcc4^−/−^* spleens (*vs*. p53^3KR/3KR^ spleens). To examine whether p53-mediated ferroptosis plays a role in testicular atrophy, we performed the similar analysis of p53-mediated activities in testis. Indeed, p53 was activated and SLC7A11 expression was significantly reduced (Figure 4F); the ferroptosis marker PTGS2 was also dramatically upregulated in *p53^3KR/3KR^Xrcc4^−/−^* testis (Figure 4G). In contrast, no obvious ferroptotic cell death was detected in the testis from either *p53^3KR/3KR^Xrcc4^+/+^* or *p53^−/−^* mice. Moreover, downregulation of SLC7A11 was also obesreved in lives, brain and bone morrows of *p53^3KR/3KR^Xrcc4^−/−^* mice although the levels of PTGS2 expression were not highly induced in those tissues, suggesting that additional factors might be required for ferroptosis induction (Figure S6). Taken together, these data demonstrate that p53-mediated downregulation of SLC7A11 is induced in the cells and tissues of *p53^3KR/3KR^Xrcc4^−/−^* mice and also suggest that p53-mediated ferroptosis may play a role in both preventing tumor development and testicular atrophy observed in the *p53^3KR/3KR^Xrcc4^−/−^* mice.

## DISCUSSION

Normal proliferating cells constantly cope with a variety of stress signals from both outside and inside such as replication stress, telomere shortening and ROS damage, and their genome integrity are inevitably damaged during proliferation, which can induce a p53-mediated temporary arrest at cell cycle checkpoints to allow cells to correct possible defects, thereby avoiding the transmission of genetic lesions to daughter cells [39]. Cell cycle arrest is released and cell proliferation resumes once the damage is repaired. Extensive and irreparable damage can trigger cellular p53-mediated senescence and apoptotic cell death. In contrast, the cell cycle progression proceeds with accumulated unrepaired DNA damage in p53-null cells, leading to mutations, chromosomal aberrations, and aneuploidy. Since p53-mediated cell cycle arrest, apoptosis and senescence are abrogated in *p53^3KR/3KR^* cells, it is not surprising that DNA damage is accumulated in these cells, leading to aneuploidy. Indeed, we showed that *p53^3KR/3KR^* MEFs exhibit higher levels of aneuploidy upon DNA damage. To corroborate this finding, we generated *p53^3KR/3KR^Xrcc4^−/−^* mice by ablating the NHEJ repair pathway and found that tissues and cells derived from *p53^3KR/3KR^Xrcc4^−/−^* mice have higher basal levels of genomic instability determined by chromosome breaks and even fusions. Our results demonstrate that p53-mediated cell cycle arrest, apoptosis and senescence are absolutely essential for its ability to control genomic integrity *in vivo*. Consistent with our earlier study [10, 36], *p53^3KR/3KR^Xrcc4^−/−^* mice do not develop spontaneous tumors, suggesting that the acetylation defective mutant retains its tumor suppression activity through activating its metabolic targets. Notably, p53 is activated in both MEFs and tissues of *p53^3KR/3KR^Xrcc4^−/−^* mice associated downregulation of Slc7a11 and p53-mediated ferroptosis is induced *in vitro* and *in vivo*. Thus, our results suggest that p53-mediated ferroptosis plays a critical role in suppressing tumorigenesis despite high levels of genomic instability in *p53^3KR/3KR^Xrcc4^−/−^* mice.

The molecular basis for the aging-like phenotypes observed in *p53^3KR/3KR^Xrcc4^−/−^* mice needs further elucidation. One common denominator of aging is the accumulation of genetic damage throughout life [40] and genomic instability has been well accepted as one of the major hallmarks of aging [41]. Consistent with this notion, several DNA repair defiency mouse models have been reported associated with accelerated aging phenotypes [42-44]. Although it is possible that the role of p53 in those aging mice is simply preventing tumor formation, a direct role of p53 in aging has been supported by several animal models. For example, by generating mice with a p53 truncation mutant encoding a carboxyl-terminal fragment called M protein, Tyner et al., showed that *p53^+/m^* mice exhibited an enhanced resistance to cancer that was accompanied by reduced longevity and early onset of a number of aging phenotypes [33]. Similar results was also obtained in a transgenic mouse model overexpressing a modestly truncated, naturally occurring isoform of p53 called p44 [34], indicating that p53 has pro-aging effects regardless of the status of genomic instability. Nevertheless, the mice with extra copies of p53 gene (super-p53), or with extra copies of the Arf gene (super-Arf), or with Mdm2 hypomorphic alleles (Mdm2^puro/Δ7-12^) have a normal longevity [45-48]. Taken together, these studies indicate that the role of p53 in aging is more complex than originally anticipated. Consistent with this notion, paradoxical roles of p53 in cellular senescence has recently been reported; several studies indicate that p53 is capable of converting senescence into quiescence by inhibiting the mTOR pathway, suggesting that p53 may also have the anti-aging activities *in vivo* [49-52]. It is very likely that only some aspects of p53 activity are activated in the mice expressing truncated p53 mutants and promote aging whereas increasing the overall function of p53 (e.g. the mice with extra copies of p53 gene or the mice with Mdm2 hypomorphic alleles) may neutralize these pro-aging effects because anti-aging activities of p53 (e.g. through inhibiting the mTOR pathway) are also enhanced in those mice.

Aging is a dynamic and complex process defined as the time-dependent functional decline. With age, homeostasis declines and damage accumulates. One of prime candidates that induce macromolecular damage is oxidative stress from reactive oxygen species (ROS) generated from normal physiological activities. Indeed, many long-lived mutants are resistant to oxidative stress [53]. Ferroptosis involves metabolic dysfunction that results in the production of both cytosolic and lipid ROS [36, 38]. Repression of *SLC7A11* transcription by p53 results in reduction of cystine uptake. Because of less cystine uptake, the levels of intracellular glutathione (GSH) will be reduced and the cellular system for defending oxidative stress is abrogated. Thus, the sensitivity of ROS-induced ferroptosis is significantly increased in p53-activating cells. We showed that SLC7A11 is downregulated by p53 and that p53-mediated ferroptosis is dramatically induced in the testis of *p53^3KR/3KR^Xrcc4^−/−^* mice. Thus, it is very likely that the combination of genomic instability and p53-mediated ferroptosis contributes significantly to the aging associated phenotypes observed in *p53^3KR/3KR^Xrcc4^−/−^* mice.

## MATERIALS AND METHODS

### Generation of p53^3KR/3KR^XRCC4^−/−^ mice

The p53^3KR/3KR^ and XRCC4^+/−^ mice described previously (Yan et al., 2006) were bred to generate p53^+/3KR^XRCC4^+/−^ mice. The p53^3KR/3KR^ XRCC4^−/−^ double knockout mice were obtained by intercrossing p53^+/3KR^XRCC4^+/−^ double heterozygous mice. The p53^3KR/3KR^ XRCC4^−/−^ offspring were PCR genotyped using primer sets (For p53^3KR^, forward: 5′-CTTCCTGCAGTCTGG GACAGC C-3′, reverse: 5′-GCAGCTGGGCCTACAG CACAC G-3′) and (For XRCC4, p1, 5′-*TAAGCTATTACTCCTGCATGGAGCATTATCACC, p2,* 5′-*GCACCTTTGCCTACTAAGCCATCTCAC and p3,* 5′-*TTCAGCTAACCAGCATCAAT AG)*. All mice work was performed in compliance with the IACUC (Institutional Animal Care and Use Committee) of Columbia University.

### Cell culture

Mouse embryonic fibroblasts (MEFs) were isolated from 13.5 day postcoital embryos obtained by heterozygous intercross according to the standard procedures. MEFs were cultured in DMEM medium supplemented with heat-inactivated FBS (MEF only) and 1% non-essential amino acids plus 200 unit/ml penicillin/streptomycin. Cells were maintained at 37°C in a 5% CO2 incubator.

### Tissue staining

immunohistochemical staining was performed using standard protocols. Tissues from mice were collected and fixed with 10% formalin overnight, then processed, paraffin-embedded, sectioned and stained with anti-mouse γ-H2ax (#2577, Cell Signaling Technologies) antibodies according to the standard procedures.

### Metaphase preparation and telomere FISH staining

The metaphase preparation and telomere FISH staining were performed as described as before with minor modifications [54]. Briefly the cells were cultured for 3 (MEFs) or 6 (bone marrow cells) hours with colcemid at the final concentration of 0.1ug/ml (GIBCO). The treated cells were then collected and swollen in 0.57% KCl hypotonic solution for 20 minutes at room temperature. After incubation, cells were fixed by adding ice-cold freshly prepared fixative (3:1 v/v methanol: acetic acid) for > 4 times. Metaphase spreads were obtained by dropping the fixed cells onto slides and steamed over the 85°C water bath for 10 sec, air-dried for > 1 hour and then checked under a microscope. For telomere FISH staining, slides were fixed in 4% (w/v) formaldehyde/PBS for 15 min at room temperature, followed by three washes with 1xPBS and digestion with pepsin (0.1%, pH was adjusted to 1.2 with HCl) (Sigma) for 10 min at 37°C. The slides were then dehydrated in ethanol after three washes with PBS. After air dry, a probe mix (70% (v/v) formamide, 2% (w/v) BSA, 100 μg/ml tRNA, 10 mM Tris, 0.5 ug/ml custom Telomere G-strand PNA probe from PNA Bio Inc) was added to each slide, and the slides were denatured by heating for 3 min at 80°C on a heat block. After 2 hours incubation in the dark wet chamber at 37°C, slides were washed twice with 70% (v/v) formamide, 0.1% (w/v) BSA, 10 mM Tris-HCl, pH 7.5, followed by three washes in 50 mM Tris-HCl, pH 7.5, 150 mM NaCl, 0.1% (w/v) BSA, and 0.1% (v/v) Tween-20. The slides were then dehydrated in ethanol serial and air dry. DNA was counterstained with DAPI. A minimum of 100 metaphases were captured and analyzed using Metafer MSearch Metaphase Finder developed by MetaSystems, MA.

### RNA isolation and qRT-PCR

Total RNA was isolated from MEFs or mouse tissues using Trizol (Invitrogen) and treated with DNase I (Ambion). 1 μg total RNA was reverse-transcribed using SuperScript III First-Strand Synthesis SuperMix (Invitrogen) following manufacturer's protocol. PCR was performed in triplicate using SYBR green mix (Applied Biosystems), and a 7500 Fast Real-Time PCR System (Applied Biosystems) under the following conditions: 15min at 95°C followed by 40 cycles of 95°C for 15 sec and 60°C for 1 min. qRT-PCR data analysis were performed as described before (Bookout and Mangelsdorf, 2003) and Primers used for RT-PCR and qRT-PCR were shown in qRT-PCR Primers.

### Flow cytometry

To analyze cell cycle by DNA content, 2×10^6^ MEFs were suspended in 200ul of PBS/0.1%FBS by vortexing after wash once with ice-cold PBS. 4mls of ice cold 70%ETOH was added to the cells one drop at a time. Cells were pelleted and resuspended in 1ml of PI solution (40ug/ml PI and 100ug/ml RNaseA) after at least 1 hr to overnight fixation at 4°C. Then FACS analysis was performed after 1 hr incubation at 37°C followed by filtering cells through 40-70um mesh. Cells were sorted using a Becton Dickinson FACScalibur machine and data were analyzed using CellQuest.

### Western blotting

Cell or tissue lysates were prepared in RIPA buffer (50 mM Tris-HCl [pH 8], 150 mM NaCl, 0.1% SDS, and 0.5% Na deoxycholate, 1% NP40 and fresh proteinase inhibitor cocktail). Protein extracts were analyzed by Western blotting according to standard protocols using primary antibodies specific for p53 (CM5, Leica Microsystems), P-S15p53 (9284, Cell Signaling Technologies), p21 (SX118, Santa Cruz), PUMA (p4743, Sigma), ATM (ab2631, Abcam), phosphor-ATM (ab81292, Abcam), Kap1 (ab10484, Abcam), phosphor-Kap1 (ab70369, Abcam), Slc7a11 (ab37185, Abcam) and β-actin (A3853, Sigma). HRP-conjugated anti-rabbit,-mouse and -rat secondary antibodies (GE Healthcare) were used and signal was detected using an ECL Western blotting detection system (GE Healthcare).

### ROS and ferroptosis inhibitor treatment

2 × 10^5^ MEF cells were seeded into 6-well plates and cultured overnight. Then cells were treated with 400 μM tert-butyl hydroperoxide (TBH, Sigma-Aldrich) to generate ROS and at the same time ferroptosis inhibitor, Ferrostatin-1 (Ferr-1, Xcess Biosciences) was added into the culture medium at the concentration of 2 μM. Cells were cultured for 8 hours, then trypsinized and stained with trypan blue followed by counting witha haemocytometer using standard protocol. Cells stained blue were considered as dead cells and cell death quantification was further confirmed by propidium iodide (PI) staining followed by FACS analysis.

### qRT-PCR primers

The following primers were used for the quantitative real-time PCR (qRT-PCR) analysis of the indicated mouse mRNAs: *β-Actin* forward 5′-GGCTGTATTCCCCTCCATCG-3′, *β-Actin* reverse 5′-CCAGTTGGTAACAATGCCATGT-3′. *Slc7a11* forward, TGGGTGGA ACTGCTCGTAAT, reverse, AGGATGTAGCGTCCAAATGC; mouse *Ptgs2* forward, GG GAGTCTGGAACATTGTGAA, reverse, GTGCACATTGTAAGTAGGTGGACT.

## SUPPLEMENTARY MATERIAL FIGURES


