# Supplementary material for: Loss of p53-mediated cell-cycle arrest, senescence and apoptosis promotes genomic instability and premature aging
Source: Oncotarget. 2016 Mar 2;7(11):11838–49. doi: 10.18632/oncotarget.7864 (PMC4914251; doi:10.18632/oncotarget.7864)
Supplement: Supplementary file 1 [file oncotarget-07-11838-s001.pdf]

Loss of p53-mediated cell-cycle arrest, senescence and apoptosis promotes genomic instability and premature aging

Supplementary Material

	Ab. MP (%)		Breaks/MP		ChrT br/MP		ChrS br/MP		Fusion/MP	
	Ave	stdev	Ave	stdev	Ave	stdev	Ave	stdev	Ave	stdev
$p53^{+/+}$	6.32	2.80	0.06	0.03	0.01	0.01	0.05	0.02	0.01	0.01
$p53^{-/-}$	17.80	0.35	0.25	0.03	0.03	0.03	0.20	0.00	0.02	0.00
$p53^{3KR/3KR}$	18.21	2.03	0.21	0.05	0.01	0.01	0.19	0.03	0.02	0.03
$p53^{-/-}XRCC4^{-/-}$	48.69	4.87	0.64	0.08	0.10	0.02	0.54	0.09	0.01	0.01
$p53^{3KR/3KR}XRCC4^{-/-}$	46.67	4.16	0.88	0.27	0.09	0.02	0.71	0.21	0.08	0.05

Supplementary Figure S1: Summary of spontaneous genomic Instability in p53 mutant MEFs

MEFs were cultured with 0.1ug/ml colcemid for 3 hours, hypotonically swollen in 0.57% KCl for 20 minutes, and fixed by ice-cold freshly prepared fixative (3:1 v/v methanol: acetic acid), then metaphase spreads were obtained by dropping fixed cells onto pre-cleaned slides. The slides were checked using a normal microscope or subjected to telomere FISH staining according to protocol described in Experimental Procedures.

A minimum of 100 cells with telomere signals were captured by Metafer4 using Metasystmes.

	Exp	MP	Ab. MP		Breaks		ChrT br		ChrS br		Fusion	
			No	Freq	No	Freq	No	Fre	No	Freq	No	Freq
**p53$^{+/+}$**	I	50	2	4.00	2	0.04	0	0.00	2	0.04	0	0.00
	II	70	3	4.29	3	0.04	0	0.00	3	0.04	0	0.00
	III	50	5	10.00	6	0.10	0	0.00	4	0.08	1	0.02
	IV	100	7	7.00	7	0.07	2	0.02	5	0.05	0	0.00
**p53$^{-/-}$**	I	50	9	18.00	13	0.24	1	0.02	10	0.20	1	0.02
	II	46	8	17.39	11	0.22	0	0.00	9	0.20	1	0.02
	III	50	9	18.00	15	0.28	3	0.06	10	0.20	1	0.02
**p53$^{3KR/3KR}$**	I	50	8	16.00	8	0.16	0	0.00	8	0.16	0	0.00
	II	59	11	18.64	13	0.22	0	0.00	13	0.22	0	0.00
	III	50	10	20.00	16	0.26	1	0.02	9	0.18	3	0.06
**p53$^{-/-}$ XRCC4$^{-/-}$**	I	42	20	47.62	30	0.71	4	0.10	26	0.62	0	0.00
	II	9	4	44.44	5	0.56	1	0.11	4	0.44	0	0.00
	III	50	27	54.00	34	0.66	4	0.08	28	0.56	1	0.02
**p53$^{3KR/3KR}$ XRCC4$^{-/-}$**	I	50	21	42.00	35	0.64	4	0.08	25	0.50	3	0.06
	II	28	14	50.00	37	1.18	3	0.11	26	0.93	4	0.14
	III	50	24	48.00	43	0.82	4	0.08	35	0.70	2	0.04

Supplementary Figure S2: Raw number of breaks and fusions from independent metaphases

Metaphase spreads for indicated MEF cells were prepared for telomere FISH staining according to the protocol described in Experimental Procedures. DNA was counterstained with DAPI and >100 cells with telomere signals were captured by Metafer4 using Metasystmes. At least 3 independent MEF lines for each genotype were analyzed for frequency of cytogenetic abnormalities specifically categorized by chromosomal breaks, chromatid breaks and chromosomal fusions.

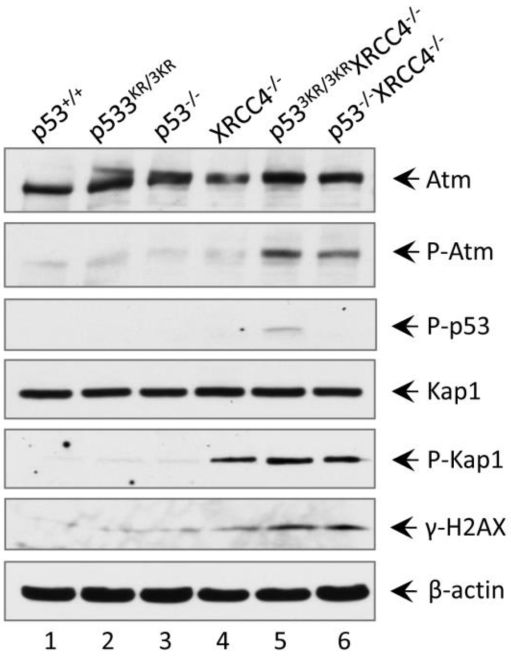

Supplementary Figure S3: The basal levels of genomic instability are increased in *$p53^{3KR/3KR}Xrcc4^{-/-}$* MEFs, related to Figure 2

Western blot analysis of the passage 1 MEFs with indicated genotypes for the expression of ATM, phosphor-ATM, Kap1, phopho-Kap1, p53, and phosphor-p53 and γ-H2ax. β-actin serves as a loading control.

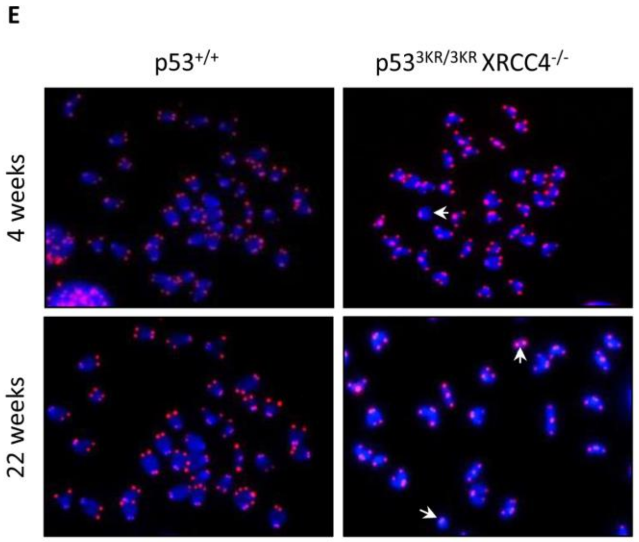

Supplementary Figure S4: Genomic instability in $p53^{3KR/3KR}XRCC4^{-/-}$ mice was increased with age, related to Figure 2

Representative images of abnormal metaphases obtained from bone marrows of mice with indicated genotypes at 4 or 22 weeks of age. Chromosomes were stained with telomere specific PNA-probes (red) and DNA was counterstained with DAPI (blue). The abnormalities were indicated by white arrows.

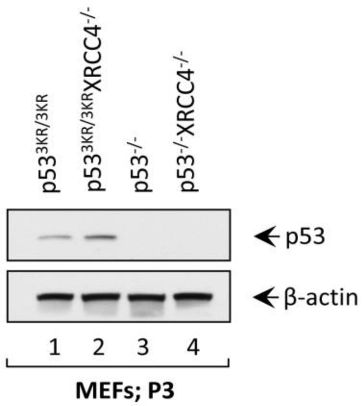

Supplementary Figure S5: *p53^{3KR}* is stabilized and activated in *p53$^{3KR/3KR}$Xrcc4$^{-/-}$* mice embryonic Fibroblasts , related to Figure 4

Western blot analysis of the passage 3 MEFs with indicated genotypes for the expression of p53. β-actin serves as a loading control.

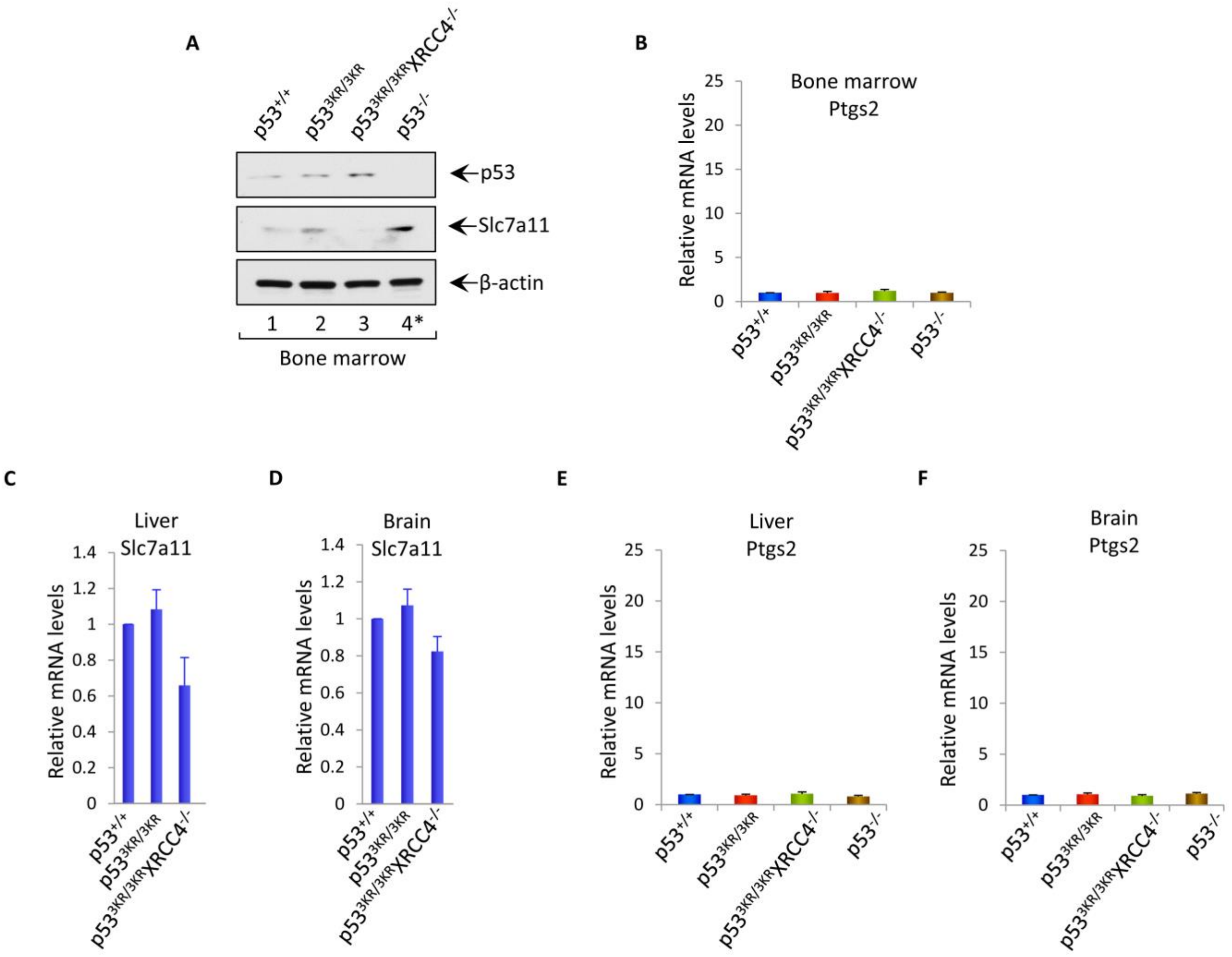

Supplementary Figure S6: SLC7A11 was downregulated in livers, brains and bone marrows of *p53$^{3KR/3KR}$Xrcc4$^{-/-}$* mice but PTGS2 was not highly induced, related to Figure 4.

A. Western blot analysis of p53, Slc7a11 and β-actin proteins in the lysates prepared from the bone marrow of 22-week-old p53$^{+/+}$, p53$^{3KR/3KR}$, p53$^{3KR/3KR}$XRCC4$^{-/-}$, and 3-month-old p53$^{-/-}$ mice at the age of 22 weeks. *; The 3-month-old tumor free p53$^{-/-}$ mice were used for control.

B. qRT-PCR analysis of Ptgs2 mRNA levels in the bone marrow (BM) of 22-week-old p53$^{+/+}$, p53$^{3KR/3KR}$, p53$^{3KR/3KR}$XRCC4$^{-/-}$ and 3 month old tumor free p53$^{-/-}$ mice. Data were shown as average ± SEM from three mice for each genotype.

C and D. qRT-PCR analysis of Slc7a11 mRNA levels in the liver (C) and brain (D) of p53$^{+/+}$, p53$^{3KR/3KR}$, and p53$^{3KR/3KR}$XRCC4$^{-/-}$ mice at the age of 22 weeks. Data were shown as average ± SEM from three mice for each genotype.

E and F. qRT-PCR analysis of Ptgs2 mRNA levels in the liver (E) and brain (F) of 22-week-old p53$^{+/+}$, p53$^{3KR/3KR}$, p53$^{3KR/3KR}$XRCC4$^{-/-}$ and 3-month-old tumor free p53$^{-/-}$ mice. Results were shown as average ± SEM from three mice for each genotype.
